# μ-Opioid receptor gene (OPRM1) polymorphism in patients with breast cancer

**DOI:** 10.1007/s13277-015-3113-z

**Published:** 2015-01-25

**Authors:** Anna Cieślińska, Edyta Sienkiewicz-Szłapka, Elżbieta Kostyra, Ewa Fiedorowicz, Jadwiga Snarska, Konrad Wroński, Michał Tenderenda, Beata Jarmołowska, Michał Matysiewicz

**Affiliations:** 1 Department of Biochemistry, Faculty of Biology and Biotechnology, University of Warmia and Mazury, Oczapowskiego 1A Street, 10-719 Olsztyn, Poland; 2 Department of General Surgery, Faculty of Medical Sciences, University of Warmia and Mazury, Wojska Polskiego 37 Street, 10-228 Olsztyn, Poland; 3 Department of Oncology, Faculty of Medical Sciences, University of Warmia and Mazury, Wojska Polskiego 37 Street, 10-228 Olsztyn, Poland; 4 Department of General and Minimally Invasive Surgery, Faculty of Medical Sciences, University of Warmia and Mazury, Niepodległości 44 Street, 11-041 Olsztyn, Poland

**Keywords:** Polymorphism, OPRM1, Breast cancer, μ-Opioid receptor, Opioids

## Abstract

Structure-dependent μ-opioid receptor (MOR) activity is an important element in cancer opioid analgesic effectiveness. It is widely accepted that guanine (G) substitution for adenine (A) at OPRM1 gene sequence position 118 changes receptor glycosylation pattern. This is associated with decreased binding ability in both exogenous and endogenous opioids, resulting in increased human pain resistance. The endogenous opioid system’s function in body homeostasis maintenance is considered mainly regulatory, so its participation in breast tumor formation and progression is identified herein. We examine the association of the most frequent MOR (A118G) gene polymorphism on breast cancer risk in a Northeastern Polish population by PCR-RFLP comparison of A and G allele frequency at OPRM1 gene A118G polymorphic site in breast cancer-diagnosed patients with healthy control group frequencies. Our results highlight a strong association between G allele presence at μ-opioid receptor A118G and increased breast cancer incidence (OR = 3.3, 95 % CI 2.2–5.0, *p* < 0.0001) and female gender (OR = 2.0, 95 % CI 1.4–2.9, *p* = 0.0004). Consequently, OPRM1 G allele presence at that site is a highly significant risk factor in breast cancer development.

## Introduction

The GLOBOCAN 2012 International Agency for Research on Cancer database shows breast cancer (BC) is the most common cancer in females worldwide, at approximately 25 % of all female cancers, and the second most commonly occurring malignancy. It ranks as the fifth major global cause of cancer death and the second in developed countries [1]. It is currently believed that tumor development in the majority of cases is associated with a genetic background, and this includes sporadic tumors [2]. This resulted from increased focus on natural gene population variability as a determinant of cancer susceptibility. At least 80 different genes with 145 variants were investigated to determine the relationship between single nucleotide polymorphisms (SNPs) and breast cancer risk, with polymorphism considered significant in approximately 35 genes with 46 variants [3]. Herein, the previously untested μ-opioid receptor gene (OPRM1) is examined as the newest candidate in this regard.

Opioids form part of the extensive physiological system of neuronal, hormonal, and immune regulation essential for bodily homeostasis maintenance. Their pain perception control and analgesia is moderated by endogenous and exogenous opioid peptide interaction with receptors situated in the central and peripheral nervous systems, in the immunologic system, and on the endothelial cell surface [4–7].

While opioid influence on tumor growth and cancer progression is less established, there is increasing evidence for its support [8–13]. μ-Opioid receptor (MOR) expression in breast cancer cells has been demonstrated in both the MCF-7 and T47D ER-positive BC cell lines and in patient biopsy specimens [14–17]. Chatikhine and coworker’s immunohistochemical studies [18] showed that the most common type of BC invasive ductal carcinomas can prove positive for β-endorphin and Met-enkephalin opioid peptides [18]. Further research has revealed that clinically relevant doses of analgesic opioids, such as morphine, promote basal cell proliferation and migration, and accompanying angiogenesis results in direct tumor growth and metastasis [9, 19, 20] or in indirect morphine-induced immunosuppression [13]. In contradistinction, however, morphine and other opioids also promote tumor cell death [15, 16, 21]. While these facts suggest that the endogenous opioid system can participate in breast cancer development and progression, many results on opioid and BC interrelationships are contradictory. One reason for this discord may be genetic variation in the OPRM1 gene.

The human OPRM1 gene is located on chromosome 6q24–q25. It spans 236 kb with at least 11 exons and 17 different splice variants under the control of multiple promoters [22]. The most commonly occurring MOR polymorphism is A118G variant (rs1799971). This is located in the first exon of gene and consists of non-synonymous substitution of guanine (G) for adenine (A), resulting in the exchange of aspartic acid for asparagine at MOR protein position 40 (N40D). This leads to loss of the N-glycosylation site in the G protein-coupled receptor’s extracellular region and associated change in its molecular level function [23]. Clinical observations have demonstrated a relationship between A118G polymorphism frequency and response to exogenous opioids, with resultant pain threshold variation [24–28]. Further studies suggest that G allele carriers exhibit decreased breast cancer-specific mortality [29] and significantly lowered risk of esophageal carcinoma development [30].

Our previous research identified opioid peptides in lactating women’s milk, where concentrations of cryptopeptides derived from human β-casein β-casomorphin-5 and β-casomorphin-7 were dependent on lactation phase [31]. This result combined with lactation as a protective factor in breast cancer risk and the abovementioned opioid participation in tumor biology prompted our examination of the relationship between the A118G polymorphism in the OPRM1 μ-opioid receptor gene and breast cancer occurrence.

## Patients and methods

### Patients and control group characteristics

Our study subjects were 151 recently diagnosed female breast cancer patients recruited at the Department of General Surgery and Oncology of the Warmia and Mazury University Hospital and at the General and Oncologic Surgery Wards of the Warmia and Mazury Oncology Centre in Olsztyn in 2013–2014. The contraindications for research qualification were breast cancer recurrence after previous surgery, metastasized cancer from other organs, and/or previous radiotherapy or chemotherapy. The control group consisted of 590 unrelated volunteers recruited on the basis of the screening at the abovementioned departments and at the University Health Center in Olsztyn who had not reported of any type of cancer, infectious disease, or surgery for at least 3 years prior to recruitment. Both experimental and control group members reported a history of no opioid drug use. All participants gave informed consent and data was collected from their medical records and/or a completed questionnaire. Each person supplied peripheral blood as research material, and all investigation procedures accorded with ethical standards and were approved by the Local Bioethics Committee (permit number: OIL.492/12/Bioet).

### Sample genotyping

Participant’s 2-mL peripheral venous blood samples were collected in Vacutainer EDTA tubes, marked with individual ID code, and transported to the laboratory in blind case-control status. Genomic DNA was isolated from whole blood using GeneJET™ Whole Blood Genomic DNA Purification Mini Kit (Thermo Scientific) according to the manufacturer’s instructions and stored at −80 °C prior to genotyping. The selection of the OPRM1 PCR reaction primers was based on Romberg et al.’s publication [32], with the following sequences: Oprm1F-5′-GGTCAACTTGTCCCACTTAGATCGC-3′ and Oprm1R-5′-AATCACATACATGACCA GGAAGTTT-3′.

PCR amplification was conducted in a thermal cycler according to the following conditions: initial denaturation at 94 °C for 3 min, followed by 40 cycles of 30 s at 94 °C complete denaturation, 30 s at 61 °C for starter attachments, 30 s synthesis at 72 °C, and final elongation at 72 °C for 5 min. The 25-μL mixture volume comprised DreamTaq™ Green Master Mix (Thermo Scientific), specific starters, the DNA matrix, and molecularly pure water (Sigma-Aldrich). The PCR product yield and specificity were evaluated by electrophoresis in 1.5 % agarose gel (Promega) stained with GelGreen (Biotium). The PCR products were then digested with FastDigest® Bsh1236I restriction enzyme (Thermo Scientific) according to the manufacturer’s instructions, separated on 2.5 % agarose gel, and identified by GelGreen (Biotium) staining. Genotyping was confirmed by the DNA sequencing of random chosen samples.

### Statistical analysis

The frequency distribution of common risk factors for BC, including age, menstrual and menopausal history, age at menarche and at first child birth, breast-feeding, family history of BC, and smoking, was examined in our study population by the Pearson *χ* ^2^ test or by the Mann–Whitney *U* test. The genotype distribution among subjects was analyzed for Hardy–Weinberg equilibrium (HWE) using the chi-square test, and genotype and SNP allele frequencies were compared in cancer patients and control groups by Fisher’s test. Odds ratios (ORs) and 95 % confidence intervals (CIs) were calculated using logistic regression analysis and used to compare both allele frequencies in cancer patients and healthy controls, and allele frequencies between female and male. The risk of breast cancer development was estimated via variant GG and AG genotype comparison with the wild-type AA homozygote. Statistical analysis was conducted on GraphPad Prism software (v 6.01; San Diego, CA, USA), with a *p* value ≤0.05 considered statistically significant.

## Results

The study population consisted of 151 histopathologically confirmed breast cancer patients and 590 healthy controls (287 female and 303 male). The distribution of selected characteristics in breast cancer females and healthy volunteers is listed in Table 1, with no statistically significant differences in the majority of compared features. All subjects were Caucasians, of approximately the same age. Both healthy and breast cancer female groups (HF and BCF, respectively) had similar fertility rates, and they reached sexual maturity, menopausal status, and gave birth at equivalent ages. Most HF and BCF were breast-fed, with a slightly less percent in the BCF group. While 10 % of both groups were nulliparous, more BC females were postmenopausal (*p* = 0.03) and more were smokers (*p* = 0.03). In addition, while both female groups, and only a few more BC than HF, reported a positive breast cancer family history, healthy males recorded BC occurrence in their female relatives twice less frequently (*p* = 0.02). Clinical evidence and histopathology classified all BCs herein invasive, with 58 % in stage I and 42 % in stage II (data not shown).

**Table 1:** Distribution of selected characteristics in breast cancer patients and healthy individuals

Characteristics	Control *n =* 590		Breast cancer female (BCF) *n =* 151	*p* value^a^ (BCF vs. HF/BCF vs. HM)
	Healthy male (HM) *n =* 303	Healthy female (HF) *n =* 287		
Age (years), mean (SD)	49.8 (12.4)	53.3 (26.2)	55.1 (10.3)	0.93/0.28
Age of menarche (years), mean (SD)	n.a.	13.3 (1.1)	13.1 (1.2)	0.35
Age of first birth (years)^b^, mean (SD)	n.a.	26.1 (3.5)	25.8 (3.5)	0.35
Number of live births^b^, mean (SD)	n.a.	1.8 (0.6)	2.0 (0.7)	0.62
Age at menopause (years)^c^, mean (SD)	n.a.	48.3 (2.4)	49.1 (3.1)	0.27
Postmenopausal status, %	n.a.	88.2	94.7	0.03
Breast-feeding^b^, %	n.a.	80.1	74.9	0.13
Nulliparous, %	n.a.	9.8	10.6	0.78
Positive family history of BC, %	8.5	13.6	17.3	0.31/0.02
Smoking, %	34.8	28.3	37.0	0.03/0.72
Ethnicity (Caucasian race), %	100	100	100	1.00/1.00

*n.a.* not applicable

^a^Two-sided Pearson χ^2^ test and Mann–Whitney test where it is appropriate.

^b^Among 135 parous BC cases and 259 parous controls

^c^Among 143 parous BC cases and 253 parous controls

The observed genotype distribution of OPRM1 A118G polymorphism in both control subgroups and cancer patients conformed to the Hardy–Weinberg equilibrium (*p* > 0.05). This suggests no population stratification and no sampling bias. The AA, AG, and GG genotypes were identified, with 0.1 G allele frequency in the entire research population. Of the total 741 participants, 609 had genotype AA, 119 had AG, and 13 had GG. These latter 13 GG genotypes were in 5 healthy individuals and 8 cancer-diagnosed patients. All healthy GG homozygous carriers were females under 50 years of age, and 3 of these were premenopausal. The AG genotype was present in 75 healthy control individuals and 44 BC patients. The respective genotype distribution and A/G allele frequencies are listed in Table 2.

**Table 2:** Genotype and allele frequencies of OPRM1 gene A118G polymorphism in studied groups and G allele breast cancer association

Research group	% of genotypes			Frequency of alleles	
	AA	AG	GG	A	G
Study population (*n* = 741)	82.2	16.1	1.7	0.90	0.10
Control group M + F (*n* = 590)	86.4	12.7	0.9	0.93	0.07
Healthy male (*n* = 303)	86.8	13.2	0.0	0.91	0.09
Studied female (BC + H) *(n* = 438)	79.0	18.0	3.0	0.88	0.12
Healthy female (*n* = 287)	86.1	12.8	1.7	0.92	0.08
*Breast cancer female (n = 151)*	*65.6*	*29.1*	*5.3*	*0.80*	*0.20*
	OR (95 % CI) GG + AG vs AA			*p* value	
*Breast cancer female vs. healthy female*	*3.3 (2.2–5.0)* ^*a*^			*<0.0001*	
Breast cancer female vs. control group M + F	3.6 (2.5–5.2)^b^			<0.0001	
Female (BC + H) vs. male	2.0 (1.4–2.9)^b^			0.0004	
Healthy female vs. healthy male	1.2 (0.8–1.9)^b^			0.42	

*CI* confidence interval, *OR* odds ratio

^a^OR adjusted for age, age of menarche and menopause, age of first birth, number of live births, breast-feeding, postmenopausal status, family history of cancer, and smoking status

^b^OR adjusted for age, family history of cancer, and smoking status

Our results suggest a strong association between the presence of the G allele at position 118 of the μ-opioid receptor gene and increased breast cancer incidence (OR = 3.3, 95 % CI 2.2–5.0, *p* < 0.0001). Significant difference was also identified in G allele occurrence in the female gender (OR = 2.0, 95 % CI 1.4–2.9, *p* = 0.0004).

## Discussion

Although the endogenous opioid system’s precise role in tumor initiation and development remains undetermined, a relationship with cancer is anticipated because it plays a regulatory function in organism homeostasis, and opioid peptides and receptors are present in cancer cells [14, 17, 18]. The identification of functionally significant genetic variation in the opioid system compounds this issue. Global reports indicate that OPRM1 A118G allele frequencies are highly dependent on ethnicity, where the overall G allelic frequency varies between 0.8 and 48 % in different ethnic groups [23]. Genotypic distribution analysis of our entire population identified 10 % G allele frequency, thus approximating the established global Caucasian frequency of 11–17 % [23].

Herein, we investigated the association between A118G OPRM1 gene polymorphism and breast cancer risk. We found that female G allele carriers had more than three times increased breast cancer risk than both healthy female and the entire control group. The one previous study on the relationship between A118G OPRM1 gene and breast cancer by Bortsov et al. recorded that breast cancer-specific mortality was significantly reduced in patients possessing the OPRM1 118G variant allele [29]. Additionally, while the protective effect of this SNP was limited only to invasive cases, it increased with increasing cancer diagnosis stage. These authors concluded this was due to reduced opioid response in patients who possessed one or both G alleles. In contrast, we examined only primary invasive breast cancer patients with no history of opioid use. The μ-opioid G variant receptor may therefore play a protective role by decreasing opioid drug action in cancer progression and metastasis, and simultaneously increase the risk of endogenous opioid peptide action in the carcinogenic process. Mura et al.’s [23] summary of research into the molecular consequences of A118G OPRM1 polymorphism indicates that this phenomenon is possible because, in contrast to the wild-type A variant N40 receptor effect, the G variant receptor can be associated with both decreased and increased signal transduction. It is important to note here that females can also be influenced by β-casomorphin-5 and β-casomorphin-7 opioid peptides in lactation milk. While both β-casomorphin peptide concentrations changed with lactation phase in healthy breast-feeding females [31], concentrations remained the same throughout lactation in the breast milk of females with food allergies [33]. While this expected time-dependent β-casomorphin gradient in healthy lactating females inspires a comparative study of the interrelationships between breast-feeding period, respective genotypes, and breast cancer diagnosis, our current study group has inappropriate stratification to pursue this herein. Future research will establish study population stratification which identifies interrelationships between the number of live births, maternal lactation duration, feeding periods, and food allergy status.

Mainstream clinical A118G OPRM1 polymorphism analysis focuses on opioid-induced analgesia and opioids, vulnerability to alcohol and nicotine addiction, and therapy response. It is interesting here that our female OPRM1 G allele frequency is approximately twice that of males. No males were GG homozygous and only 40 carried one G allele. Some studies indicate gender-specific associations [34–36], and Fillingim et al.’s allelic distribution of tested SNP in healthy people specifies only females [28]. Although these authors’ population was more ethnically diverse than ours, their G allele overrepresentation in females is consistent with our results. This may constitute one explanation why breast cancer is so rare in males; accounting for only 0.6 % of all breast cancer patients worldwide [37]. It is acknowledged that male BC does not significantly differ from female BC, except in obvious gender-specific differences, and also that breast cancer is more likely to be estrogen receptor (ER)-positive in males than in females [38]. It should be noted that functional relationship between ERα and MOR has been reported recently. It was shown that MOR activation leads to ERα activation and mediates its translocation to the plasma membranes. The synergistic induction of breast cancer cell proliferation has been observed as the final effect [39]. Moreover, it has also been shown that expression of ERβ can be regulated by opioids [40]. In conclusion, since the MOR G allele (40D variant) proves more sensitive to endogenous opioid peptides and promotes the recently reported estrogen receptor interaction, our results highlight the significance of the OPRM1 G allele as a serious risk factor in breast cancer development.
